# Metabolic Remodeling during Long-Lasting Cultivation of the *Endomyces magnusii* Yeast on Oxidative and Fermentative Substrates

**DOI:** 10.3390/microorganisms8010091

**Published:** 2020-01-09

**Authors:** Elena P. Isakova, Irina N. Matushkina, Tatyana N. Popova, Darya I. Dergacheva, Natalya N. Gessler, Olga I. Klein, Anastasya V. Semenikhina, Yulia I. Deryabina, Nicola La Porta, Nils-Eric L. Saris

**Affiliations:** 1 A.N. Bach Institute of Biochemistry, Research Center of Biotechnology of the Russian Academy of Sciences, Leninsky Ave. 33/2, 119071 Moscow, Russia; ddarya1993@gmail.com (D.I.D.); gessler51@mail.ru (N.N.G.); klein_olga@list.ru (O.I.K.); yul_der@mail.ru (Y.I.D.); 2 Department of Medical Biochemistry and Microbiology, Biology and Soil Science Faculty, Voronezh State University, Universitetskaya pl.,1, 394000 Voronezh, Russia; biomed-popova@yandex.ru (I.N.M.); tpopova@bio.vsu.ru (T.N.P.); semenikhina@bio.vsu.ru (A.V.S.); 3 IASMA Research and Innovation Centre, Fondazione Edmund, Mach, Department of Sustainable Agroecosystems and Bioresources, Via Mach 1, 38010 San Michele all’Adige, Italy; 4 Department of Food and Environmental Sciences, University of Helsinki, Viikki Biocenter 1, POB 56, 00014 Helsinki, Finland; n-e.saris@zitran.com

**Keywords:** *Endomyces magnusii*, mitochondria, glutathione system, yeast, antioxidant enzymes, reactive oxygen species, aging

## Abstract

In this study, we evaluated the metabolic profile of the aerobic microorganism of *Endomyces magnusii* with a complete respiration chain and well-developed mitochondria system during long-lasting cultivation. The yeast was grown in batches using glycerol and glucose as the sole carbon source for a week. The profile included the cellular biological and chemical parameters, which determined the redox status of the yeast cells. We studied the activities of the antioxidant systems (catalases and superoxide dismutases), glutathione system enzymes (glutathione peroxidase and reductase), aconitase, as well as the main enzymes maintaining NADPH levels in the cells (glucose-6-phosphate dehydrogenase and NADP^+^-isocitrate dehydrogenase) during aging of *Endomyces magnusii* on two kinds of substrates. We also investigated the dynamics of change in oxidized and reduced glutathione, conjugated dienes, and reactive oxidative species in the cells at different growth stages, including the deep stationary stages. Our results revealed a similar trend in the changes in the activity of all the enzymes tested, which increased 2–4-fold upon aging. The yeast cytosol had a very high reduced glutathione content, 22 times than that of *Saccharomyces cerevisiae*, and remained unchanged during growth, whereas there was a 7.5-fold increase in the reduced glutathione-to-oxidized glutathione ratio. The much higher level of reactive oxidative species was observed in the cells in the late and deep stationary phases, especially in the cells using glycerol. Cell aging of the culture grown on glycerol, which promotes active oxidative phosphorylation in the mitochondria, facilitated the functioning of powerful antioxidant systems (catalases, superoxide dismutases, and glutathione system enzymes) induced by reactive oxidative species. Moreover, it stimulated NADPH synthesis, regulating the cytosolic reduced glutathione level, which in turn determines the redox potential of the yeast cell during the early aging process.

## 1. Introduction

A gradual accumulation of various detrimental changes, which together increase the possibility of disease and ultimately result in death, is the main cause of aging. In 1956, Denhan Harman [1] put forward the free radical theory of aging, claiming that the gradually accumulated harmful effects of reactive oxygen species (ROS) coupled with endogenous metabolic reactions were responsible for the aging and death of all living things [2]. Senescence is reported to be associated with increased ROS generation while the activity of antioxidant cell systems declines. Both effects lead to dramatic impairment of mitochondrial functions and cell physiology in general [2]. According to the free radical theory of aging, a rise in the rate of metabolic processes (mainly respiratory enzyme activity) causes a high intensity of free radical reactions and results in high levels of intracellular ROS, giving rise not only to aging processes but also to the development and evolution of life, genetic defects, and various illnesses of organisms [3]. Thus, according to the free radical theory of aging, activation of ROS generation due to an increase in the metabolic rate is the dominant factor in longevity. The detrimental effects of ROS generated during the free radical reaction are minimized by ROS scavenging enzymes, such as catalases (CATs), superoxide dismutases (SODs), and the glutathione antioxidant system [4,5]. Superoxide anion radical is a predecessor for most ROS and a mediator of oxidative reactions [4]. Free radical dismutation is induced by SODs producing H_2_O_2_, which is reduced to H_2_O by some CATs and peroxidases via redox features of haem groups in the enzyme [4]. Besides, eukaryote cells contain glutathione and thioredoxin systems together with some low-molecular-weight compounds capable of deactivating ROS and maintaining the cell redox state [6,7]. However, an imbalance between ROS generation and the capacity of the cell to neutralize them (under unfavorable conditions or due to the aging process) can induce hyper-oxidation and oxidative stress, which causes damage to the biological macromolecules, and leads to a pathological cellular state and programmed cell death (apoptosis) [8,9]. Little is known about the response of aconitate hydratase (AH) and some of the other TCA cycle enzymes to increased generation of oxygen radicals, including the process of aging [10]. Age-related changes in the activity of citric acid cycle enzymes may potentially affect cellular bioenergetics and redox status. O_2_^−^ and H_2_O_2_ do not appear to directly inhibit AH, instead they require an interaction between AH and a membrane component responsive to peroxide [10]. There is no measurable alteration in the age-related activity of NAD^+^-isocitrate dehydrogenase, the isozyme that functions in the citric acid cycle. However, mitochondrial NADP^+^-isocitrate dehydrogenase (IDH) shows a modest age-related increase in activity, which would probably result in increased mitochondrial NADPH levels. However, the NADPH/NADP^+^ ratio usually decreases with age, which may be associated with an age-related increase in the pro-oxidant state in mammalian mitochondria, also demonstrated by a reduction in the glutathione reduced to glutathione oxygenized (GSH/GSSG) ratio [11].

Corroborated by numerous experiments, the free radical theory of aging has made an essential contribution to understanding the aging process in various organisms. However, data emerged in the 2000s showing an absence of any correlation between low metabolism levels (low metabolic rate) and increased longevity in nematodes and insects [12,13]. Moreover, restricting calorie intake by between 10% and 50% unexpectedly facilitated the metabolic rate (oxygen consumption and heat production) in *Caenorhabditis elegans* [12]. Lower eukaryotes and yeasts are excellent tools for studying cell aging at the single cell level [13]. Two models of aging using yeast cultures have been proposed: replicative lifespan (RLS), which is defined by the number of daughter cells produced by a mother cell before senescence; and chronological life span (CLS), defined as the period in which non-dividing cells remain alive. The study of yeast cells has shown beyond doubt that ROS accumulate during the so-called diauxic transition (the shift to the stationary growth phase) and the stationary growth phase [13]. It has also been reported that nutrient limitation in the yeast population, particularly of glucose and amino acids, triggers an increase in both RLS and CLS [14]. Mitohormesis has been found to play a role in regulating stress resistance and enhancing lifespan in some organisms and this has been studied in yeast cells. Calorie restriction in yeast cells results in increased H_2_O_2_ production at the stationary growth stage, which promotes SOD activity and extends CLS [15]. Superoxide anion-radical generation at the stationary stage inhibits ROS generation, and also extends CLS [16]. Calorie restriction could upregulate the mitochondrial proteome and respiratory functions, while the altered mitochondrial proteome induces mitohormesis. Most of the changes occur during the switch from glycolysis to respiration (i.e., the diauxic shift) [17]. Expression of the proteins participating in oxidative phosphorylation is therefore transformed into lifespan [16]. Aging and apoptosis have been shown to be closely related in yeasts, although the nature of this relationship has yet to be elucidated. The nature of the relationship between antioxidant enzyme induction and lifespan also remains unclear. The overexpression mutants in cellular detoxifying enzymes have a longer CLS but the relationship is non-linear [18]. For example, overexpression of SOD2 (Mn-dependent mitochondrial SOD) did not change the lifespan of mice, but it enhanced the resistance of them to prooxidant paraquate [19].

Current data concerning the various aspects of yeast aging are somewhat controversial and do not allow us to draw any conclusions regarding the mechanisms underlying lifespan regulation. So far, all the results on the effects of various metabolic types on lifespan have been obtained using the facultative aerobe *S. cerevisiae*, which prefers glucose and other fermentable sugars (in particular, galactose) as their main carbon source. The composition of the respiratory chain of the mitochondria in traditional yeast species, namely *Saccharomyces cerevisiae*, *Candida utilis* is quite variable. In particular, the mitochondria of *S. cerevisiae* have no complex I, and in the *C. utilis* organelles, complex I is induced only in the stationary growth phase under the conditions of limited nutrients [20]. By contrast, the *E. magnusii* yeast possesses invariable complex I and the absence of alternative pathways of respiration. This feature of mitochondria allows the yeast to utilize successfully the substrates of oxidative type, namely glycerol, lactate, ethanol, while most species of the *S. cerevisiae* gender usually assimilate only the substrates of fermentable type. According to the above-mentioned statement, we consider that the *E. magnusii* species is a unique model, similar in its mitochondrial system to the animal ones. It can be convenient and easy to work with this yeast studying the mechanisms of aging.

We sought to establish how the functioning of the antioxidant systems, the adaptive defense response, and possible pathological processes under oxidative stress during yeast cell aging interact with each other. The results could also shed new light on the dynamics of the activity of key enzymes regulating the cell’s redox state in the yeast *E. magnusii*.

## 2. Materials and Methods

### 2.1. Yeast Strain and Culture Conditions

The *E. magnusii* yeast was grown in batches of 100 mL in glycerol—(1%) and glucose—(1%)-containing media of the following composition (g/L): MgSO_4_—0.5, (NH_4_)_2_SO_4_—0.3, KH_2_PO_4_—8.6, NaCl—0.1, CaCl_2_—0.05, yeast extract—2.0, *L*-histidine—0.003, *L*-methionine—0.003, and *L*-tryptophan—0.003 at 28 °C as described previously [21]. Absorbance was assessed in cell suspension at the wavelength of 590 nm (A_590_) using a Specol-11 spectrophotometer (Carl Zeiss, Oberkochen, Germany). Cells were harvested at different stages of growth: logarithmic (A_590_ = 2.6–2.7), early stationary (24 h of growth, A_590_ = 4.0–4.1), late stationary (48 h of growth, A_590_ = 4.5–4.6), deep stationary 1 (96 h of growth, A_590_ = 4.4–4.7), deep stationary 2 (168 h of growth, A_590_ = 4.4–4.7).

### 2.2. Cell Viability and Vitality Assays

To determine cell viability, the yeast cells from different growth phases were centrifuged at 3500× *g* for 10 min at 4 °C, washed with sterile water, and suspended to the final density of 10^8^ cells mL^−1^ in 100 mM phosphate buffer with pH 7.0. Yeast cells were suspended in phosphate-buffered saline (PBS), and a 200 µL sample of the cell suspension was mixed with 100 µL methylene blue (0.1 mg mL^−1^ stock solution, dissolved in a 2% dihydrate sodium citrate solution) and incubated for 5 min at room temperature. Viability was examined under a light microscope using Gorjaev’s chamber (×400) from at least 1000 cells in one biological replicate. Viable cells were colorless, and dead ones were blue [22]. For the spotting test, cells were suspended in sterile water and diluted 10^−6^, 10^−5^, or 10^−4^ cells mL^−1^. Samples (10 µL) of each suspension were inoculated on solid YPD (medium contained (g/L): yeast extract 10.0, peptone–20.0, dextrose–20.0, agar–20.0), pH 5.5, and incubated at 28 °C for two days. Colony growth was inspected after 48 h.

### 2.3. Cell Respiration

Oxygen consumption by the yeast cells was assessed in vitro at +25 °C using electrodes covered by a fluoroplastic film at a constant potential of 660 mV. The incubation medium for the experiment contained 50 mM KP_i_; pH 5.5, and 1% glucose [23].

### 2.4. Potential-Dependent Staining

Potential-dependent staining of mitochondria in the *E. magnusii* cells raised in the different growth phase by rhodamine (Rh123). Cells were incubated with 0.5 µM Rh123 and examined in 0, 15, 20, and 30 min. Incubation medium contained 0.01 M PBS, pH 7.4; 1% glycerol or glucose, respectively. Regions of high mitochondrial polarization are indicated by red fluorescence due to the concentrated dye. To examine the Rh123-stained preparations, filters 02, 15 (Zeiss, Oberkochen, Germany) were used (magnification × 100). Photos were taken using an AxioCam MRC camera.

### 2.5. Transmission Electron Microscopy (TEM)

The TEM analysis of untreated *E. magnusii* yeast cells was performed as described previously [23]. Briefly, the yeast cells were raised in the logarithmic growth phase, precipitated, fixed with 2.5% glutaraldehyde in 0.1 M sodium phosphate buffer (pH 7.2) for 2 h, and then post-fixed in 1% OsO4 for an hour at room temperature. After dehydration, the samples were embedded in Epon 812. Ultrathin sections were prepared with an LKB-8800 ultratome using diamond knives. Thereafter, the sections were stained with uranyl acetate for 60 min and post-stained as described previously, and examined with a Jeol (JEM-100B) and Hitachi U-12 electron microscopes (Hitachi, Tokyo, Japan).

### 2.6. Preparation of Cellular Homogenate

The cellular homogenates from the cells raised in different growth phases were obtained according to the standard protocol as described previously [24]. Cells were twice washed with ice-cold water, centrifuged at 3500× *g* for 10 min, and disrupted with mortar using liquid nitrogen for 2 min. The obtained homogenate was resuspended in grinding medium (1:5 *w*/*v*). The medium contained 20 mM KP_i_, pH 7.2; 2 mM EDTA, 0.5 mM phenyl-methylsulfonyl-fluoride (PMSF). Then the homogenate was centrifuged at 10,000× *g* for 30 min. The supernatant was used for the experiments. Cellular proteins were assayed by Bradford method [24] with BSA as standard.

### 2.7. Antioxidant Enzymes Activities Assay

Total CATs activity was assessed by monitoring the cellular homogenate at 240 nm with an Sf-2000 spectrophotometer (Spectr, Sankt Peterrburg, Russia). The procedure was performed in 20 mM KP_i_ buffer, pH 7.2; containing 2 mM EDTA. The reaction was triggered by the application of H_2_O_2_ (the molar extinction coefficient 46.3 M^−1^ cm^−1^). The CATs activity was measured by monitoring the decrease in H_2_O_2_ [25]. A_240_ was determined every 15 s for 3 min after the reaction start. CATs activity was expressed in µmol used H_2_O_2_ × min^−1^ × mg^−1^ of protein. The activity of SODs in cell suspension was assessed by monitoring of A_406_ with a Specol-11 spectrophotometer (Carl Zeiss, Oberkochen, Germany) upon inhibition of quercetin autooxidation as described in [26]. The procedure was performed in 20 mM KP_i_ buffer; pH 10.2, containing 0.8 mM TMDA, 0.1 mM EDTA, and 1.4 µM quercetin. Fifty percent inhibition of quercetin autoxidation was considered as a SOD enzymatic activity unit.

### 2.8. AH, Glucose-6-Phosphate Dehydrogenase (G6PDH), and IDH Activity Assays

Cellular AH activity was measured in 50 mM Tris-HCl-buffer, pH 7.8; containing 4 mM citrate by monitoring the decrease in the absorbance of A_235_ due to the formation of cis-aconitate with a double bind for 3 min. The enzyme amount catalyzing 1 µmol reaction product per min at 25 °C was considered as one unit of enzyme activity [27].

### 2.9. Cellular IDH Activity

Cellular IDH activity was measured in 50 mM Tris-HCl-buffer, pH 7.7; containing 1.5 mM isocitrate, 2 mM MnCl_2_, 0.25 mM NADP^+^, 0.1 mM EDTA by monitoring the increase in the absorbance resulting from NADP reduction due to the conversion of isocitrate into α-ketoglutarate catalyzed by the enzyme at 340 nm. The amount of the enzyme catalyzing 1 µmol α-ketoglutarate per a 1 min at +25 °C is considered as one enzyme activity unit. The number of enzyme units (E) is calculated using the formula:

E = ∆D × V_c_ × V/∆V × T × 6.22 (1)

where ∆D is the decrease in A_340_ for a certain period time; V_c_ is the volume of solution in the cuvette, mL; V is the total volume of enzyme solution, mL; ∆V is the amount of the sample, mL; τ–the time of measurement, min; 6.22–extinction coefficient, the change in absorbance at 340 nm upon reduction or oxidation of 1 µmol coenzyme [28].

### 2.10. G6PDH Activity

G6PDH activity was assayed in 0.05 mM Tris-HCl-buffer, pH 8.0; containing 1 mM 6-phosphogluconate and 0.12 mM NADP^+^ by measuring the increase in absorbance resulting from the reduction of NADP^+^ at 340 nm. One unit of enzyme (EU) activity is that amount of enzyme, which reduces 1 µmol NADP^+^ per min at +25 °C. The calculation is the same as that for IDH.

### 2.11. Assay of Enzyme Activities of Glutathione Antioxidant System

#### 2.11.1. Cellular Glutathione Peroxidases (GPxs)

Cellular glutathione peroxidases (GPxs) were measured at 340 nm in 0.05 mM K-P_i_-buffer, pH 7.4; containing 1 mM EDTA, 0.12 mM NADP^+^, 0.85 mM reduced glutathione (GSH), 0.37 mM H_2_O_2_, and 1 unit of glutathione reductase (GR) per mL. The same medium without glutathione served as the control. The reaction was initiated by the enzyme application. The rate of NADP^+^ reduction due to enzyme reactions such as oxidized glutathione formation by GPxs action followed by its reduction resulted from NADP^+^ oxidation by GR action was monitored by decrease in A. One unit of enzyme (EU) activity was defined as the enzyme amount catalyzing 1 µmol final reaction product at +25 °C per a min. The calculation is the same as that for IDH.

#### 2.11.2. Cellular GR Activity

Cellular GR activity was assayed by monitoring the decrease in the absorbance due to NADPH oxidation which resulted from glutathione reduction by GR. Cellular suspension (1–5 × 10^8^ cells) was added into 1 mL 0.05 M K-Pi-buffer, pH 7.4; containing 1 mM EDTA, 0.16 mM NADPH, 0.8 mM GSSG. The calculation is the same as that for IDH.

### 2.12. Glutathione HPLC-ECD Analysis

The harvested yeast cells were briefly washed with deionized water and frozen in liquid nitrogen for further HPLC analysis. Then, 100 µL of re-frozen yeast lysate was added to 500 µL of 0.1 M cold iced perchloric acid (PCA). After brief vortexing, the suspension was sonicated for 5 s and placed on ice for 10–15 min for better metabolite extraction. The obtained cell homogenate was twice centrifuged for 20 min at 14,000 rpm in precooled (+4 °C) microcentrifuge. Then, 200 µL of clear supernatant was loaded into the HPLC vial for direct HPLC analysis. The HPLC system for glutathione measurements was equipped with CouloChem-III electrochemical detector, Waters 717 plus auto-sampler with a cooled platform (+4 °C), and Waters 515 HPLC pump. Then, 20 µL of mobile phase (0.1 M LiH_2_PO_4_, 1.5 mM octenyl succinic anhydride, and 7% methanol) was delivered at a flow rate of 1.0 mL/min in an isocratic mode. Pre-column was Super ODS A0114; 4.6 mm × 5 cm, particle size 2 µm, and analytical column was ESA HR-80; 80 mm × 4.6 cm, P/N 68-0100 with particle size 3 µm and pore size 120 A. The both columns were maintained at +30 °C. Under the conditions, GSH eluted at 1.61 min and GSSG did at 2.72 min. Samples processing, file storage, and data analysis were controlled by EZChrom Elite software. The concentration of GSH and GSSG was calculated based on a calibration curve equation per mg of protein.

### 2.13. Detection of ROS

The dynamics of intracellular ROS production was monitored using a spectroscopic fluorescence probe of dihydro-2′,7′-dichlorofluorescein diacetate ester (H_2_DCF-DA) (Sigma, Saint-Luis, MO, USA) as described previously [29]. Cells were harvested at different stages of growth: logarithmic (A_590_ = 2.6–2.7), early stationary (24 h of growth, A_590_ = 4.0–4.1), and late stationary (48 h of growth, A_590_ = 4.5–4.6), deep stationary 1 (96 h of growth, A_590_ = 4.4–4.7), deep stationary 2 (168 h of growth, A_590_ = 4.4–4.7).

### 2.14. Assay of Diene Conjugation

To estimate total cellular conjugated dienes (DC), the lipids from cellular homogenates were extracted by the mixture of heptane and isopropyl and centrifuged at 4000× *g* for 10 min. The supernatant was mixed with some distilled water (1:10), stirred twice, and the heptane phase was dissolved in ethanol (1:10). The spectra of ultraviolet absorption scans between 220 and 300 nm were measured using the Beckman DU-8 spectrophotometer. The process is accompanied by the appearance of the maximum in the area of 232–234 nm and a shoulder in the area of 260–280 nm, which matched coupled ketodienes [30]. A molar extinction coefficient of 2.2 × 10^5^ M^−1^ s^−1^ cm^-l^ was used for the calculation of the conjugated dienes [31].

### 2.15. Statistical Analysis

The experiments were performed in biological triplicates with a standard error of less than 5%. The influence of pH and temperatures on soluble carbohydrates and lipids was estimated using one-way ANOVA (*n* = 3) with R (R Core Team 2016). The Student *t*-test was used to analyze the significance of differences for independent samples. Values were considered significant at *p* < 0.05.

## 3. Results

### 3.1. The Growth of Yeast Cultivated on Two Substrates

**Figure 1:**
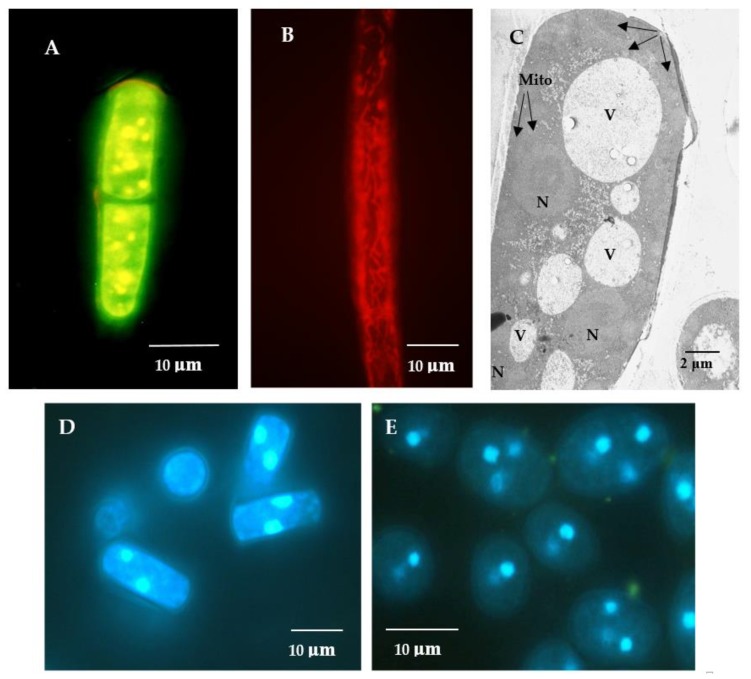
Potential-dependent staining of mitochondria in the *E. magnusii* cells raised in the logarithmic growth phase by JC1 (**A**), Rh123 (**B**). (**D**,**E**)—the cells were raised in the logarithmic (**D**) and late stationary stages (**E**) and labeled with 0.3 μM DAPI for DNA. **A,B**—the cells were incubated with 0.5 μM JC1 or Rh123 for 20 min. The incubation medium contained 0.01 M phosphate-buffered saline (PBS), 1% glycerol, pH 7.4. The areas of high mitochondrial polarization are indicated by bright-yellow (**A**), bright-red (**B**) fluorescence due to the concentrated dye. To examine the Rh123-stained preparations, filters 02, 15 (Zeiss) were used (magnification 100×). Photos were taken using an AxioCam MRc camera. C—ultrastructure of the *E. magnusii* cells after 30 min incubation. N—nucleus, Mito—mitochondria, V—vacuole. (**C**)—ultrastructure of the *E.magnusii* cells. N—nuclei, V—vacuoles, Mito—mitochondria.

**Figure 2:**
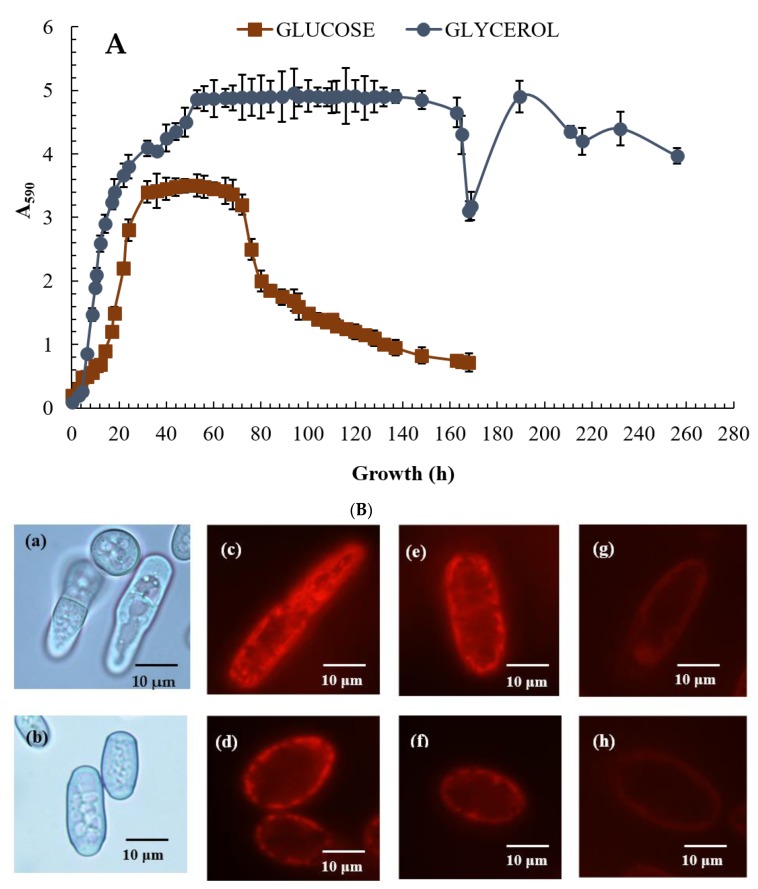
Growth curves of the *E. magnusii* yeast (**A**) and cell energy status (**B**) on glycerol- and glucose-containing media. (**A**)—absorbance was assessed every two hours in cell suspension at the wavelength of 590 nm (A_590_) using the spectrophotometer. The presented data are the mean of three independent cultivations (with comparable results) each in triplicate with the standard deviation. (**B**)—micro-images of the *E. magnusii* yeast in the logarithmic growth phase in glycerol–(**a**) and glucose–(**b**)-containing media. (**B)**, (**c**–**h**)—potential-dependent staining of the *E. magnusii* cells raised in the different growth phases with Rh123. The cells were incubated with 0.5 µM Rh123 and examined after 0, 15, 20, and 30 min. Incubation medium contained 0.01 M phosphate-buffered saline (PBS), 1% glycerol or 1% glucose, pH 7.4. The regions of high mitochondrial polarization are bright-red due to concentrated dye. (**Ba**,**c**,**e**,**g**)—glycerol-containing media; (**Bb**,**d**,**f**,**h)**—glucose-containing media. (**c**,**d**)—logarithmic phase; (**e**,**f**)—96 h of growth; (**g**,**h**)—168 h of growth. To examine the Rh123-stained preparations, filters of 02, 15 (Zeiss) were used (magnification × 100). Photos were taken using an AxioCam MRc camera.

The *E. magnusii* yeast is a polynuclear fungus (Figure 1C–E) capable of forming the pseudomycelium and the true mycelium. The *E. magnusii* yeast with a strongly-pronounced aerobic metabolism possesses a well-developed membrane apparatus with abundant sophisticated mitochondria with numerous cristae (Figure 1A,B). The growth curves of the yeast *E. magnusii* differed significantly according to the different types of substrate used. Growth on 2% glycerol, an “oxidative” substrate, led to stable biomass accumulation for about 20 h up to the stationary growth stage and continued during that phase, which lasted a further 16 h, i.e., for a total of 36 h (Figure 2A). There was then a slight rise in biomass accumulation in the 36–53 h growth stage followed by steady stationary growth throughout the experiment with a non-significant (20%) transitive decrease at around 168 h of growth (Figure 2A). The experiment was conducted over 20 days and there was no significant deviation in the stationary phase (data not shown). The pattern of growth was very different with the “fermentative” substrate (1% glucose). The maximum biomass yield was almost half that with glycerol (Figure 2A), while the stationary phase was much shorter (no longer than 40 h) and was followed by irreversible culture lysis leading to significant fall in biomass yield by 168 h of cultivation (Figure 2A).

The obtained results prompted us to study the energy status and survival of the yeast *E. magnusii* at various growth stages, namely at 12–16 h, 24 h, 48 h, 96 h, and 168 h of cultivation. They were considered as the logarithmic, early stationary, stationary, late stationary, and deep stationary phases, respectively. Besides, we assayed the same parameters for the transition phase into the decrease in growth (in the case of glycerol) and degradation of the cell culture phase (in the case of glucose).

### 3.2. Respiratory Activity of E. magnusii Yeast Cultivated on Different Substrates

The assay of the respiratory activity of the yeast *E. magnusii* showed it to be generally higher in the cells grown on the oxidative glycerol than those grown on glucose (Table 1). At the logarithmic growth stage, the respiratory rate of the cells on glycerol was 40% higher than that of the cells grown on glucose (Table 1). In the early stationary growth stage, the respiratory activity of the cells cultured on glucose was higher than that of the cells grown on glycerol, but the difference (about +18%) was not significant. However, the tendency seen in the logarithmic stage persisted during further growth and reached its maximum after 168 h (Table 1). The assay of cyanide-resistant oxidase activity showed that it was induced in the cells grown on glycerol in the stationary growth phase. In the culture grown on glucose, the alternative oxidase was induced at the late stationary stage (Table 1). The cyanide-resistant pathway made its most significant contribution (about 50%) to total cellular respiration in the late stationary phase (48 h of growth) in the cells grown on glycerol. But in the cells grown on glucose, this occurred only after 96 h (Table 1). However, it is worth noting that the highest alternative oxidase activity was observed at the 96-h growth stage in both cases, decreasing thereafter from 50.0% to approximately 31.9% on glycerol and 38.4% on glucose.

### 3.3. Potential-Dependent Staining of Mitochondria in the E. magnusii Cells

Potential-dependent staining of mitochondria in the *E. magnusii* cells raised in the different growth phases by Rh123 are shown in Figure 2B. As it is seen in fluorescent microimages (magnification × 100), the intact mitochondria in the energized *E. magnusii* cells from the logarithmic growth stage using both substrates are bright red and mainly rod-shaped (Figure 2B,c,d). It indicates high energy cellular potential under these conditions. Rh123 fluorescence intensity declined while the culture was growing, significantly decreasing by 168 h of growth (Figure 2B,g,h). The data well agreed with the obtained results on dynamics of the respiratory activity of the *E. magnusii* cells at various growth stages (Table 1).

### 3.4. The Survival of Yeast Cultivated on Different Substrates

**Figure 3:**
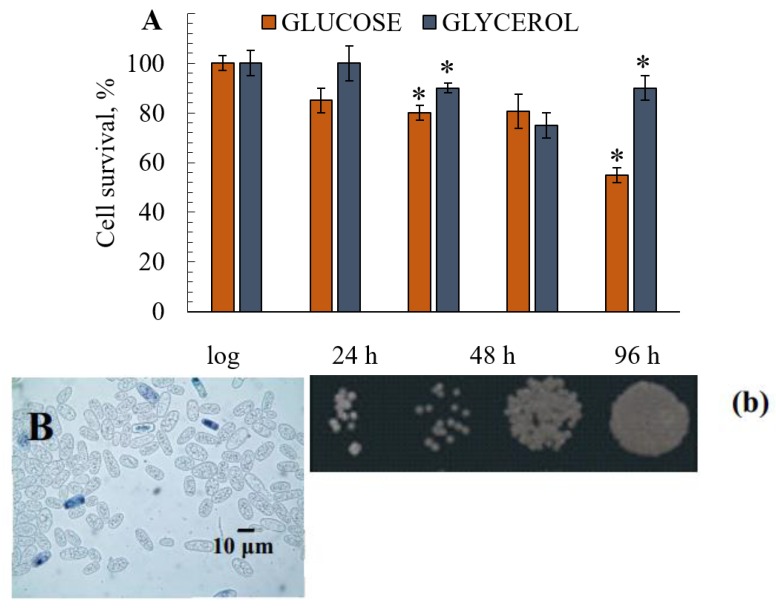

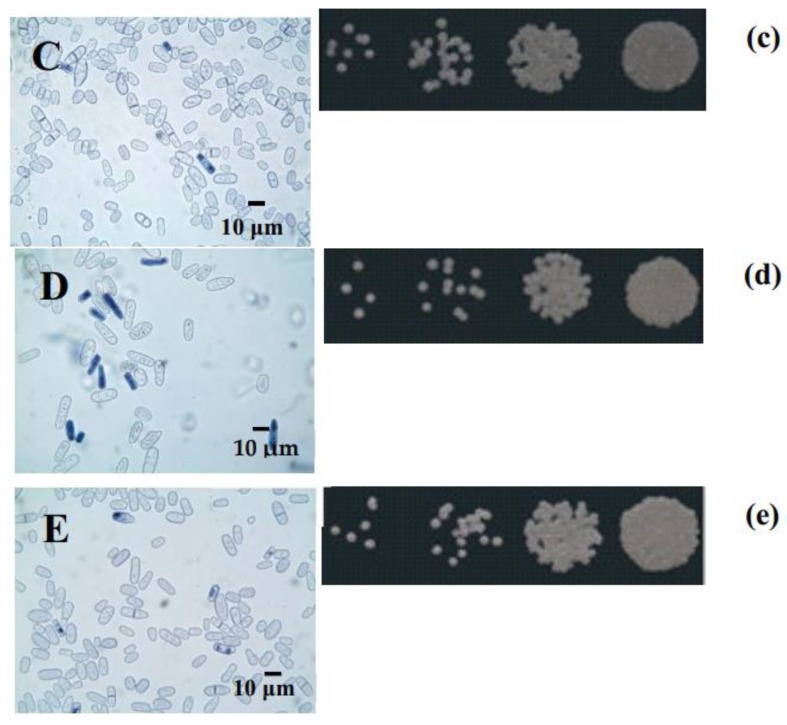
The survival of the yeast grown in glycerol- and glucose-containing media (**A**). The results are presented as mean values ± SEM (n = 4). * Differences between glucose-utilizing and glycerol–utilizing cells were statistically significant (*p* < 0.05). (**B**–**E**)—micro-images of the *E. magnusii* yeast at 96 (**B**,**C**) and 168 (**D**,**E**) hours of growth in glycerol—(**C**,**E**) and glucose—(**B**,**D**)-containing media. For cell viability, the yeast cells from different growth phases were centrifuged, washed with sterile water. Yeast cells were suspended in PBS, and a 200 µL sample of the cell suspension was mixed with 100 µL methylene blue and incubated for 5 min at room temperature. Viability was examined under a light microscope using Gorjaev’s chamber (×400) from at least 1000 cells in one biological replicate. Viable cells were colorless, and dead ones were blue. (**b**–**e**)—Spot dilution assays in YPD media containing glucose (**b**,**d**) or glycerol (**c**,**e**) (2 µL per spot) for the *E. magnusii* yeast raised at 96 (**b**,**c**) and 168 (**d**,**e**) hours of growth.

During the 7-day experiment, the growth of the culture grown on 1% glycerol was not inhibited (data not shown). The percentages of budding cells varied from 17% at the logarithmic growth stage to 5%–6% after 168 h of growth, showing that the cell ability to bud was not suppressed (Figure 3C,c). The percentages of budding cells in yeast cultivated on 1% glucose were in the range of 15%–18% in the logarithmic growth phase and decreased to 1%–2% by the 168-h cultivation phase that cannot compensate the number of lysing cells (Figure 3D,d). The assay of *E. magnusii* showed that survival decreased as the culture aged, regardless of the substrate. However, when the culture was grown on glucose, the survival of the remaining cells in the suspension began to decrease after 24 h of growth and had nearly halved after 168 h of growth (Figure 3A,D,d). On glycerol, the survival rate fell slightly after 48 h of cultivation and had decreased by no more than 10%–15% at the end of the experiment (7 days; Figure 3B,E,e).

### 3.5. ROS Generation and DC Accumulation

**Figure 4:**
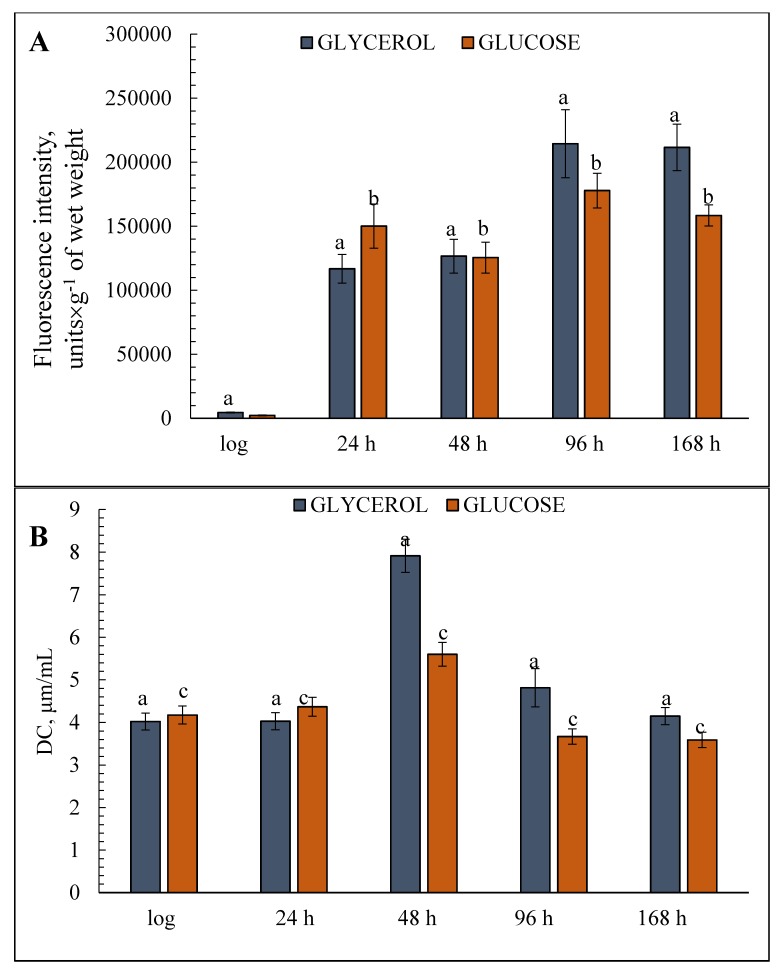
Estimates of total cellular diene conjugates (DCs) and reactive oxygen species (ROS) level in the glycerol—(**A**) and glucose—(**B**) assimilating *E. magnusii* yeasts, raised at different growth stages. The dynamics of intracellular ROS production was monitored using a spectroscopic fluorescence probe of dihydro-2′,7′-dichlorofluorescein diacetate ester H_2_DCF-DA. Total cellular DCs were extracted by the mixture of heptane and isopropyl and centrifuged at 4000× *g* for 10 min. The supernatant was mixed with some water, the heptane phase was dissolved in ethanol. The spectra of ultraviolet absorption scans between 220 and 300 nm were measured using the Beckman DU-8 spectrophotometer with the appearance of the maximum in the area of 232–234 nm and a shoulder in the area of 260–280 nm. A molar extinction coefficient of 2.2 × 105 × M^−1^ × s^−1^ cm^−l^ was used for the calculation of the DCs. The incubation medium for the experiments contained 50 mM KPi, pH 5.5; and 1% glucose. Values are mean ± S.E.M from 5–6 independent experiments. The intensity of ROS production in the version of «positive control» made up 234195.83 fluorescence intensity, units. a, b, c—0.05 > *p* > 0.005.

ROS generation and the accumulation of initial products of lipid degradation (DCs) were examined to assess the initial level of peroxyl products and their final level resulting from the destructive effects of ROS. The lowest ROS level was found at the logarithmic growth stage under both cultivation conditions. The H_2_-DCFDA fluorescence intensity, which indicates the ROS level, in the cells grown on glycerol was almost double that in the cells grown on glucose (Figure 4). However, this trend changed later on and the ROS level jumped sharply at the early stationary stage in the cells grown on both substrates (25-fold on glycerol, 65-fold on glucose) with further increases in both cases at 48 and 96 h of cultivation (Figure 4). ROS generation in the cells reached its maximum on glycerol and was about 20% higher than on glucose. The assay of DCs accumulation revealed similar tendencies in the cells on both substrates, i.e., a two-fold increase in the DCs content at the late stationary stage (48 h) followed by a decrease to the initial level (Figure 4). The DCs baselines were more or less comparable under both growth conditions (Figure 4).

### 3.6. Analysis of Antioxidant Enzyme (CATs and SODs) Activity in the Yeast E. magnusii

**Figure 5:**
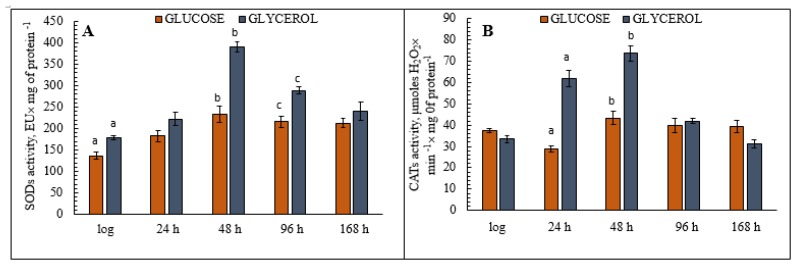
The assay of the total cellular superoxide dismutases (SODs) (**A**) and catalases (CATs) (**B**) activities in the *E. magnusii* yeast. **A**—The activity of SODs in cell suspension was assessed by monitoring of A_406_ with a spectrophotometer upon inhibition of quercetin autooxidation. The procedure was performed in 20 mM KP_i_ buffer, pH 10.2; containing 0.8 mM TMDA, 0.1 mM EDTA, and 1.4 µM quercetin. 50% inhibition of quercetin autoxidation was considered as a SODs enzymatic activity unit. Values are mean ± SEM from 5–6 independent experiments. **B**—Total CATs activity was assessed by monitoring the cellular homogenate at 240 nm with a Specol-11 spectrophotometer (Germany). The procedure was performed in 20 mM KPi buffer, pH 7.2, containing 2 mM EDTA. The reaction was triggered by the application of H_2_O_2_ (the molar extinction coefficient 46.3 M^−1^ cm^−1^). The CATs activity was measured by monitoring the decrease in hydrogen peroxide concentration. A_240_ was determined every 15 s for 3 min after the reaction start. Catalase activity was expressed in µmol-used H_2_O_2_ × min^−1^× mg^−1^ of protein. Error bars represent the standard deviation of triplicates to each experiment. a, b—*p* < 0.05, c—did not differ significantly.

The assays of the activity of key antioxidant enzymes during aging of the yeast *E. magnusii* grown on glycerol showed that activity increased significantly at the late stationary growth stage (48 h) (Figure 5A). At this stage, SODs activity in those cells had increased by 70% compared to the logarithmic growth stage, then it remained at almost the same level for the rest of the experiment (Figure 5A), while total activity in the cells grown on glucose increased 1.6-fold in the late stationary phase but then there followed a clear, gradual decrease up to 168 h of cultivation (Figure 5A). Cellular CATs activity during aging displayed the same tendency as SOD activity under both conditions (Figure 5B), but it peaked in the cells grown on glycerol at a level 1.7 times higher than that of the cells grown on glucose.

### 3.7. Assay of the Activity and Level of the Glutathione System Enzymes

**Figure 6:**
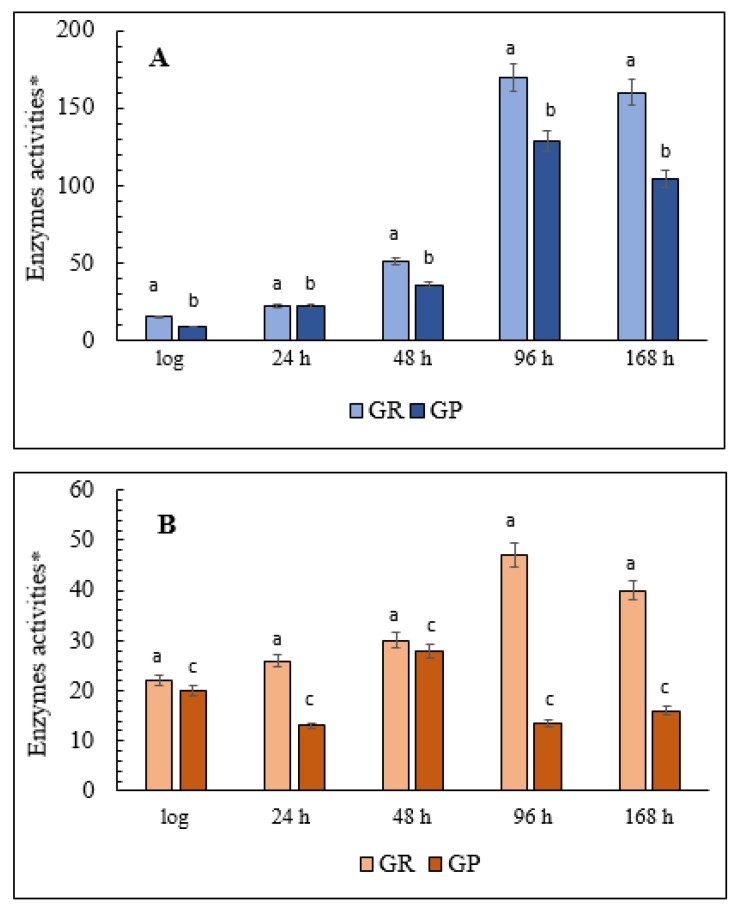
The estimates of the total cellular glutathione system enzymes activities in the *E. magnusii* yeast. (**A**)—glycerol-; (**B**)—glucose-containing media. * Unit of enzymatic activity per 1 mg of protein. Cellular GPxs were measured at 340 nm in 0.05 mM K-P_i_-buffer, pH 7.4; containing 1 mM EDTA, 0.12 mM NADP^+^, 0.85 mM GSH, 0.37 mM H_2_O_2_, and 1 unit of GR per mL. The same medium without glutathione served as the control. The reaction was initiated by the enzyme application. The rate of NADP^+^ reduction due to enzyme reactions such as oxidized glutathione formation by GPxs action followed by its reduction resulted from NADP^+^ oxidation by GR action was monitored by decrease in A. * One unit of enzyme (EU) activity was defined as the enzyme amount catalyzing 1 µmol final reaction product at +25 °C per a min. The calculation is the same as that for isocitrate dehydrogenase (IDH). Values are mean ± SEM from 5–6 independent experiments. Error bars represent the standard deviation of triplicates to each experiment. a, b—*p* < 0.05; c, d—*p* < 0.04.

The changes in the activity of the glutathione system enzymes, including glutathione peroxidase (GPxs) and glutathione reductase (GRs), differed in the cells grown on the two substrates. The assay of the glutathione system in the cells grown on glycerol showed that by 168 h of cultivation, GPxs activity had increased more than 14-fold and GRs more than 10-fold compared with the logarithmic growth phase (Figure 6A). Also, GPxs activity slightly increased in the deep stationary (96-h) growth phase (Figure 6A). In the cells grown on glucose, the levels of GPxs and GRs activity increased gradually and reached minor peaks at the 48- and 96-h cultivation stages, respectively (Figure 6B). However, even the highest levels of activity of these enzymes were less than a third of those in the cells grown on glycerol (Figure 6B).

### 3.8. Glutathione Content in the Yeast E. magnusii

**Figure 7:**
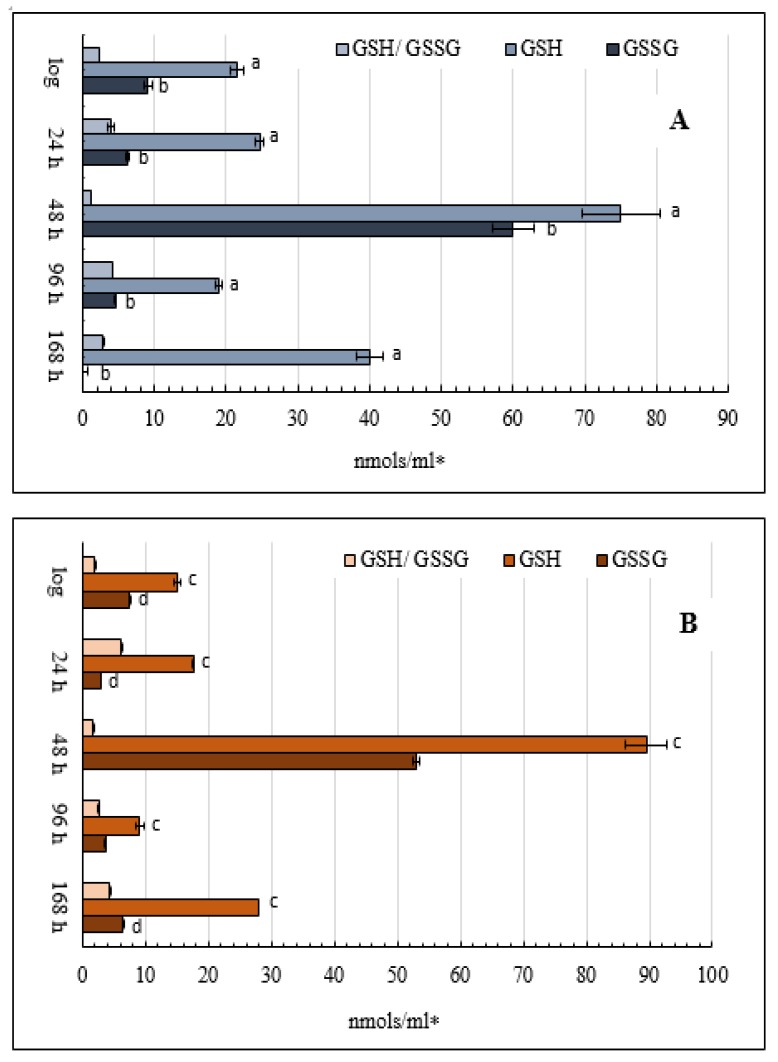
Estimates of total cellular oxidized and reduced glutathione in the glycerol—(**A**) and glucose—(**B**) utilizing *E. magnusii* yeast in different growth stages. The harvested yeast cells were briefly washed with deionized water and frozen in liquid nitrogen for further HPLC analysis. 100 µL of re-frozen yeast lysate was added to 500 µL of 0.1 M cold iced PCA. The obtained cell homogenate was twice centrifuged for 20 min at 14,000 rpm in precooled (+4 °C) microcentrifuge. Then, 200 µL of clear supernatant was loaded into the HPLC vial for direct HPLC analysis. Values are mean ± SEM from 5–6 independent experiments. Error bars represent the standard deviation of triplicates to each experiment. a, b, c, d—*p* < 0.05.

The reduced glutathione level (GSH) was about 1.5 times higher in the cells grown on glycerol than in the cells grown on glucose in the logarithmic and early stationary phases (Figure 7). On glycerol, the GSH level increased about three-fold in the late stationary growth phase (48 h) compared to the logarithmic phase, and on glucose, that increased five-fold (Figure 7). The noticeable rise in the GSH level was more evident in the cells grown on glucose (Figure 7B), followed by a significant decrease up to 96 and 168 h of cultivation (Figure 7). The ratio of GSH to GSSH had two peaks under both conditions: in the early and deep growth stationary phases.

Since the NADPH level is closely connected with glutathione regeneration by the GRs-GPxs system, we examined the activities of the enzymes that are the main producers of the coenzyme in the yeast cell.

### 3.9. The Activity of Enzymes Serving as the Main Producers of NADPH in the Yeast Cell

**Figure 8:**
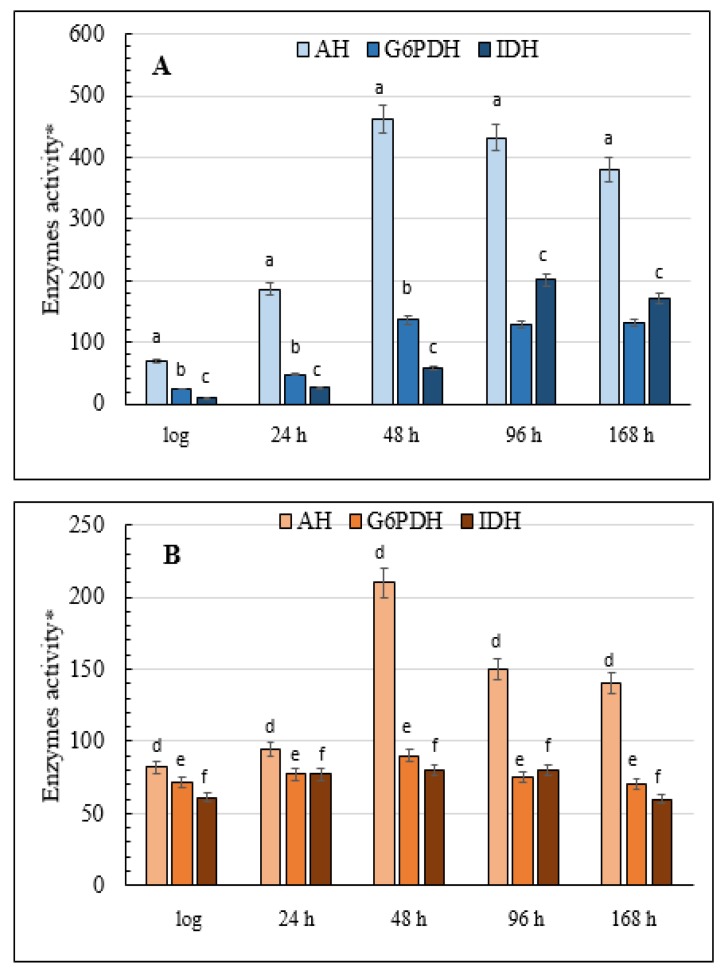
Total cellular AH, G6PDH, and IDH activities in the *E. magnusii* yeast. (**A**)—glycerol-; (**B**)—glucose-containing media. * Values are a mean unit of enzymatic activity per 1 mg of protein × 10^4^. Cellular IDH activity was measured in 50 mM Tris-HCl-buffer; pH 7.6–7.8; containing 1.5 mM isocitrate, 2 mM MnCl_2_, 0.25 mM NADP^+^, 0.1 mM EDTA by monitoring the increase in the absorbance resulting from NADP reduction due to the conversion of isocitrate into *α*-ketoglutarate catalyzed by the enzyme at 340 nm. The amount of the enzyme catalyzing 1 µmol *α*-ketoglutarate per a 1 min at +25 °C is considered as one enzyme activity unit. G6PDH activity was assayed in 0.05 mM Tris-HCl-buffer; pH 8.0; containing 1mM 6-phosphogluconate and 0.12 mM NADP^+^ by measuring the increase in absorbance resulting from the reduction of NADP^+^ at 340 nm. One unit of enzyme (EU) activity is that amount of enzyme, which reduces 1 µmol NADP^+^ per min at +25 °C. The calculation is the same as that for IDH. Values are mean ±SEM from 5–6 independent experiments. Error bars represent the standard deviation of analytical triplicates to each experiment. a–d—*p* < 0.05, e, f—did not differ significantly.

Glucose-6-phosphate dehydrogenase (G6PDH), which is the main coenzyme producer in the glutathione system, and NADP^+^-dependent isocitrate dehydrogenase (IDH) is the focus of this part of the study. The results showed that in the cells grown on glycerol, G6PDH activity gradually increased during the aging of the yeast culture, increasing six-fold up to 168 h of growth compared with the logarithmic growth phase (Figure 8A). There was a non-significant change in the level of G6PDH activity in the yeast grown on glucose, although in the logarithmic phase it was almost three times higher than in the cells grown on glycerol (Figure 8B). The activity of another key NADP-producing enzyme, IDH, in the glycerol-assimilating culture also increased more than 18-fold by the 96-h stage of the experiment compared with its initial level in the logarithmic growth phase (Figure 8A). IDH activity in the culture using glucose showed a similar trend but it increased only 1.5-fold, non-significantly by the 96-h stationary phase before returning to the initial level at the end of the experiment (Figure 8B). However, the initial activity of IDH in this condition was seven times greater than in the cells grown on glycerol (Figure 8).

### 3.10. Total Activity of Cellular AH

The significant changes we found in DC content and the activity of the key enzymes of oxidative metabolism, namely G6PDH and IDH, during aging of the yeast *E. magnusii* prompted us to analyze the activity of another important enzyme, AH, a marker of redox status and cell energy. The results showed that in the late stationary growth phase (48 h) the activity of the enzyme increased more than six-fold in the cells grown on glycerol and two-fold in the cells grown on glucose (Figure 8). Moreover, enzyme activity was not suppressed, even at the end of the experiment, and AH activity was generally comparable in the cells grown under the two conditions (Figure 8).

## 4. Discussion

In this study, we first assessed the biochemical features describing the redox status of the *E. magnusii* yeast during aging. We used the *E. magnusii* yeast, which, being an obligate aerobe, possesses the complete respiratory system similar in its mitochondrial system to the animal ones. It makes the yeast strain a unique model for studying the mechanisms of aging. The yeast with a fermentative metabolism type such as *S. cerevisiae* has an ill-developed membrane and mitochondrial apparatus, only scanty large mitochondria with small cristae. This yeast mitochondria have much fewer cristae and trend to irregularity in their shape, structure, and packing. However, the yeast with a strongly-pronounced aerobic metabolism, namely *E. magnusii*, possesses well-developed membrane apparatus with abundant complex mitochondria with numerous cristae (Figure 1). We used two different types of substrates, namely the fermentative glucose and the oxidative glycerol. One advantage of using glycerol as a carbon source is its higher degree of reduction compared to glucose. To exploit this reducing power for the production of reduced compounds thereby significantly increasing maximum theoretical yields, the electrons derived from glycerol oxidation must first be saved in the form of cytosolic NAD(P)H [32]. Glycerol is metabolized in mitochondria, so the well-developed system of mitochondria in *E. magnusii* allows to assimilate glycerol more efficiently. The *E. magnusii* yeast can consume the oxidative substrates more completely than the fermentative ones resulting in much more efficient biomass yield [33,34].

We found interesting experimental evidence demonstrating regularities in the various processes in aging (stationary) cells grown on two different substrates. Firstly, *E. magnusii* yeast grown on a fermentative substrate of 1% glucose displayed a lower biomass accumulation, a prolonged logarithmic growth phase, and a much shorter (less than 40 h) stationary phase than when grown on an oxidative substrate (Figure 2A).

Since in the majority of similar studies on *S. cerevisiae* yeast grown using different glucose concentrations (0.5% to 2%), we did the same using the *E. magnusii* yeast. The results showed that the species can grow rather well on glucose-containing media but worse than using the similar concentrations of glycerol [Supplementary]. Pan Y et al. [16] found that the wild-type yeast *S. cerevisiae* had a 40% survival rate by the seventh day of cultivation. Some studies have shown that yeast culture in the stationary growth phase can survive for a long time without additional nutrients and can maintain a survival rate of about 100% for three months when grown in a glucose-containing medium using stock compounds such as glycogen and trehalose [35]. Although in our experiments we used a 1% rather than a 2% glucose substrate in the abovementioned medium, this does not explain the low growth and early phase of culture destruction when grown on glucose. As the *E. magnusii* culture is a classic aerobe, it has a complete respiratory chain with invariable couple point I and well-developed mitochondria. Glucose is not as suitable substrate for the species as the respiratory substrates of glycerol, lactate, and ethanol, which in turn are not the preferred carbon sources for the classic fermentative yeast of *S. cerevisiae* [36,37].

Moreover, the genetic determinants of the *UBR2* and *GUT1* genes, which play a crucial role in the species’ ability to consume glycerol, have been described for bakers yeast [38]. In this case, it may have to do with a similar adaptation of respiratory yeasts to a fermentative substrate [39]. This is consistent with data on the survival of culture grown on glucose, which gradually decreases from 85% in the early stationary growth phase to 55% after 168 h of cultivation compared with the stable, high percentage of live cells in a culture grown on glycerol (Figure 1A). Secondly, regardless of the type of substrate, the DC concentration in the cells increased at the deep stationary growth phase (48 h) and O_2_^−^ generation rose in the 96 and 168-h cultivation phases (Figure 4), which shows that the hyper-oxidation state develops under both conditions. The ROS level in the cellular cytosol under normal conditions (temperature, glucose, and oxygen content) is usually very low [40]. Various studies have shown that ROS generation can be induced by the apoptosis process during aging [41]. In the culture grown on the oxidative phosphorylation substrates (2% glycerol), mitochondria are the main sources of cellular ROS due to the activity of the respiratory chain complexes (mainly complexes I and III) [42,43]. The jump in the DC level could be explained by the activity of mitochondrial complexes of electron transfer resulting in increased ROS production. An increase in the DC content of yeast cells grown on a fermentative substrate, which does not induce oxidative phosphorylation, comparable to the use of glycerol, may be related to metabolic readjustments in the diauxic shift phase. This is a growth phase in which the cell metabolism switches from fermentation to respiration, and growth on non-fermentable substrates is slow allowing the microorganisms to live on stock carbohydrates [16,44]. Confirmation of this comes from data on respiratory activity, which show an oxygen consumption rate comparable to that on glycerol, and induction of alternative cyanide-resistant oxidase in the stationary growth phase (Table 1).

The respiratory activity of the yeast *E. magnusii* along with the ROS level was much higher when the culture was grown on glycerol than when grown on glucose, especially in the early and late stationary growth phases (Table 1). Moreover, the high induction of the alternative pathway of electron transfer and its peak occurred in the cells in the 96-h growth phase with both substrates (Table 1). The results are in agreement with the data on ROS generation dynamics, where the maximum was observed in the same growth phase (Figure 4). We may, therefore, suppose that the high ROS level in our experiments led to the induction of the cyanide-resistant alternative pathway of electron transfer, which is an element of the antioxidant system [45,46].

Finally, aging yeast grown on a respiratory substrate exhibited an increase in the activity of CATs and SODs (Figure 3), the cell’s key antioxidant enzymes protecting it against oxidative stress [47,48]. Therefore, the jump in antioxidant enzyme activity in the late stationary growth phase matched the increase in DC and ROS levels (Figure 4). This tendency was revealed when we analyzed the changes in mitochondrial membrane permeability during the growth and aging of the yeast *E. magnusii* [30]. In our studies, we found that the total cellular CATs activity gradually increased during culture growth and reached its maximum in the late stationary phase (48 h) at twice that in the logarithmic phase. The activity of the other key free radical scavenging enzyme, SODs, was similar. Like CATs activity, SODs activity reached its maximum in both the mitochondria and the cellular homogenates in the late stationary phase. The powerful induction of key antioxidant enzymes in the cell (CATs and SODs) is most likely the result of an increase in ROS generation.

Besides, we observed an increase in the activity of some coupled enzymes of the glutathione system, namely GR and GPxs, which was much more significant with the glycerol substrate (Figure 6). The phenomenon can be explained by the combined activity of CATs and SODs, which are the first targets of ROS, and the glutathione system, mainly GPxs, which detoxify hydroperoxide-containing substrates via GSH. Moreover, FAD-dependent GR plays a role in reducing GSSG into GSH in the process [49]. Nicotinamide adenine dinucleotide phosphate (NADPH) is the terminal electron donor of the system. The GPxs family contains eight selenium-dependent and non-selenium enzymes (GPx1–8), which catalyze the reduction of organic hydroperoxides and their alcohols via GSH [50]. A lack of GR leads to mortality in *Sshizosaccaromyces pombe* [51]. As GR/GP system activity is highly dependent on GSH concentrations, we speculate that the explanation for the stable GSH content of the yeast cells grown on both glycerol and glucose lies not only in the induction of GPxs but also in the regeneration of the GSH GR system, which maintains the redox balance in the cell. This seems reasonable if we take into account the high respiratory activity of glycerol-utilizing yeast cells in the late stationary growth phase resulting in significant ROS production [30]. The GSH level in *E. magnusii* yeast grown in a glycerol-containing medium was 1.4–2.4 times higher than in the cells grown on glucose (Figure 7). This may be of great importance for redox-related processes, which depend on the level of GSH, which participates in signaling and transcriptional factor activity. Glutathione acts as an internal cell antioxidant by serving as a trap for free radicals, and as a co-substrate in peroxide detoxification. It plays a role in reducing the amount of Grx needed for bisulfite restoration [52]. Moreover, GSH is known to play a protective role at apoptosis induction in yeast. According to the current opinion, if GSH decreases below a certain threshold, it is a signal for apoptosis, triggered either by induction of the death receptor or by mitochondrial apoptosis signaling. In contrast, an increase in the GSH level provides cell protection against Fas-induced apoptosis. Numerous data indicate the vital role of GSH in protecting cells against the effects of various apoptotic stimuli since cell redox imbalance due to both GSH oxidation and GSH export triggers apoptosis [53]. It is worth noting that the GSH content reached its peak in the late stationary growth phase when the activity of both CATs and SODs increased. GSH is known as a good acceptor of OH^•^ radicals [54] and an increase in GR/GP system activity along with CAT and SOD activity, providing a stable GSH content in the cytosol, is probably a powerful antioxidant defense mechanism that works in response to an increase in ROS generation during aging of the yeast.

The NADPH level in the cells is another factor impacting on the activity of the GR/GP system [55]. The content of the latter in the cytosol depends on the activity of the pentose phosphate pathway and, in particular, its first enzyme, NADP-G6PDH [56], and on the activity of some alternative NADPH-producing enzymes, such as NADH-related IDH [57]. Oxidation of isocitrate into 2-oxoglutarate catalyzed by NADP-IDG is closely connected with the enzymatic transformations of GSSG and GSH (2GSH = GSSG + 2H^+^) catalyzed by GR. In turn, NADPH-dependent G6PDH triggers glucose-6-phosphate conversion into 6-phosphogluconon-Δ-lactone. G6PDH is believed to play a significant role in the survival of animal cells and in preventing ROS-induced cell death [58]. However, there are insufficient data on the changes in G6PDH activity in yeast cells during aging.

We found that growth of the yeast *E. magnusii* on glycerol was accompanied by an increase in the activity of both G6PDH and NADP^+^-dependent IDH (Figure 8A), confirming data obtained from animals. The high demand for NADPH for the functioning of the glutathione system was probably responsible for the tendency obtained in our experiments. It is worth noting that G6PDH- and NADP^+^-dependent IDH activity in the logarithmic phase of growth were, respectively, three and seven times higher on glucose than on glycerol (Figure 8B), possibly as a result of active glucose metabolism in the yeast cells. We also observed an increase in AH activity in the aging culture, with a clear jump in activity in the late stationary growth phase (Figure 8). AH is known to catalyze the reversible isomerization of citrate into isocitrate. Some cytoplasmic and mitochondrial AHs differ in their physicochemical and structural features [59]. The iso-enzymes of AH catalyzing the same reaction are assumed to perform different physiological functions in cellular metabolism, which reflect the impact of the enzymes on oxidative and biosynthesis processes. The well-known fact that O_2_^−^ inhibits AH activity suggests that this enzyme is a sensitive and crucial target for ROS action under oxidative stress [60]. An increase in AH activity in the late stationary growth phase (48 h) may be associated with an increase in cell respiratory activity, which, in turn, reflects the activation of cellular metabolism in general. Our experiments revealed the induction of some TCA enzymes in the cells in the late stationary growth phase (data not shown). In addition, the increase in AH activity may be related to its role in citrate metabolism and glyoxylate cycle activity in the cytoplasm, and in regulating the translation and stability of some mRNA by binding to them [61]. In the yeast *S. cerevisiae*, the knockout in the *ACO1* gene (Δaco1), which encodes the AH enzyme, leads to mitochondrial DNA disorders [62], which suggests that AH directly interacts with DNA and maintains its stability [63].

In this regard, the increase in AG activity in our experiments may be induced by the decrease in O_2_^−^ in the late stationary (48 h), which was much more pronounced in the case of glycerol (470 EU) compared to that on glucose (210 EU). It, on the one hand, may be considered a marker of the initial stages of oxidative stress, and, on the other hand, a defense response, indicating the poly-functionality of the enzyme. The gradual decrease in the AH level up to 48 h of growth (Figure 6), most likely induced by a significant increase in the ROS level during this phase (Figure 4), is rather interesting and is lent support by the well-known fact that mitochondrial AH is highly sensitive to ROS, much more so than the cytosolic isoform [61].

The stationary phase of yeast growth is traditionally used as a model of chronological aging and allows us to assess the lifespan of non-dividing cells [64]. The decrease in activity of the PKA, TORC1, and Sch9p pathways prolongs both chronological and replicative lifespans (CLS and RLS) [65]. It occurs, in particular, because of cell protection against oxidative stress facilitated by induction of the *SOD2* gene, which encodes mitochondrial SOD through the transcription factors Msn2p/Msn4p and Gis1p [65]. The yeast culture’s transition into the stationary growth phase and the duration of this phase are regulated by signaling and transcriptional mechanisms, which are good tools for transiting the cells to death. Comparing the results of the redox status of *E. magnusii* yeast grown on various types of substrates in the stationary phase, we can conclude that aging of the culture grown on 1% glycerol triggers powerful ROS-induced protective mechanisms (CATs, SODs, the glutathione system) and promotes NADPH synthesis, which supports cytosol GSH levels and provides high energy potential and survival of the yeast cells in the aging culture. When grown on the fermentative glucose without inducing an effective electron transfer system in the mitochondria, we saw a general decrease in the energy and antioxidant state of the cells and a much lower survival rate than when grown on the non-fermentable substrate. The role of glucose as a pro-aging carbon source has been repeatedly reported and is beyond question [65]. Glucose fermentation leads to ethanol synthesis, which is toxic to yeast cells and negatively impacts on the lifespan of the population, seemingly resulting in a dramatic fall in growth at the 80-h cultivation phase (Figure 1A) and a significant decline up to 50% in survival in the deep (168 h) stationary growth phase (Figure 2A,b). The late stationary stage (48 h of cultivation) can be considered the starting point of all metabolic readjustments when certain simultaneous processes, such as a sharp jump in ROS generation, DCs content, and key enzyme activity (CATs and SODs), are followed by enhancement of the glutathione system and NADPH-generating enzymes. When the yeast is grown on a respiratory substrate, these events significantly change its survival rate and inhibit growth, with concurrent cell degradation when the yeast is grown on glucose. The effect of hormesis may explain the differences in growth and life features of yeast cultivated under different conditions. Hormesis has recently been identified as a biphasic dose-related adaptive response, which is stimulated by low doses of stress and inhibited by high doses [66].

A currently prevailing hypothesis holds that ROS generated in the oxidative phosphorylation system in the eukaryotic mitochondria at all levels, from yeasts to humans, plays a crucial role in regulating lifespan [18]. According to this hypothesis, Krebs cycle intermediates, such as α-ketoglutarate, and the mitochondrial ROS regulate the activity of transcription factors, for example, Hif1 α, leading to readjustment of the cellular metabolism. However, key metabolic pathways impact on ROS levels and antioxidant signaling, thereby mutually regulating the lifespan [18]. There are two lines of thought regarding the adaptive role of mitochondrial ROS in the signaling increasing the lifespan of yeast. The first proposes that yeast species with reduced target of rapamycin(TOR) signaling produce high levels of mitochondrial ROS during growth, for example, superoxide anions that lead to a decrease in ROS levels in the stationary growth phase and an increased lifespan [18]. mtROS-induced hormesis and increased longevity include promoting the anti-stress response via Msn2/4 and Gis1, and triggering non-canonical induction of DNA damage response systems [43]. The second suggests that catalase is inhibited, indirectly increasing the amount of hydrogen peroxide-inducing SODs, which block superoxide anions [17]. Although there is no evidence showing which type of ROS serves as the key signal, the data confirm the coordinating role of mitochondrial ROS in stress resistance [67]. Applying the main lines of thought of this hypothesis to our case, we would expect to see CATs activity inhibited and SODs activated. However, when the yeast was grown on glycerol, we observed little, but stable, CATs activity with no sign of inhibition, and increased activity of the second SODs enzyme (Figure 3B). SOD activity is five-fold higher on glycerol than that on glucose. The growth on glycerol, modeling the conditions of calorie restriction, which in yeast cells results in increased H_2_O_2_ production at the stationary growth stage, which is reported to promote SOD activity and extend CLS [17]. The high activity of the glutathione and coupled NADPH generation systems may have taken over the CAT function (Figure 3 and Figure 4). According to the above-mentioned hypothesis, we can assume that the high CLS of the cells grown on the respiratory substrate observed in our experiments is a result of the hormetic effect of ROS produced by the mitochondrial electron transfer chain, which is active under these conditions. The hypothesis can also explain the high activity of the antioxidative defense enzymes CATs, SODs, and the glutathione system, coupled with the NADPH synthesis system, which we observed in the experiments using glycerol. Although the speculation regarding hormesis partly disproves Harman’s well-known theory, it sheds light on our results and on the data reported by a number of other authors [68]. However, there may be an alternative explanation. Glycerol is known to be assimilated in a yeast cell in three different ways: (1) into glycerol-3 phosphate through glycerol kinase reaction (*GUT1*); (2) into dihydroxyacetone through the reaction of HAD-dependent glycerol dehydrogenase; (3) into glyceraldehyde through the reaction of HADP-dependent glycerol dehydrogenase [31]. All the pathways can lead to the synthesis of triacylglycerides, which, in turn, may prolong lifespan [34]. Some authors suggest that the phenomenon could be related to the cumulative effect of the compounds, which is highly important once nutrients are depleted in the stationary growth phase. Moreover, triacylglycerides preventing lipotoxicity maintain lipid homeostasis and ensure the high chronological and replicative potential of yeast.

In conclusion, the redox and energy statuses of yeast during chronological aging can vary significantly depending on the substrate, which determines metabolic and intracellular signaling readjustments.

## Figures and Tables

**Table 1:** Respiratory activity and alternative oxidase induction of the *E. magnusii* yeast in different growth phases.

Growth Phase	Respiration Rate, ng-Atom Consumed O per 1 mg of Dry Weight *			
	Control		+KCN, 4 mM	
	Glycerol	Glucose	Glycerol	Glucose
Logarithmic phase	43.8 ± 2.3 ^a^	26.24 ± 3.01 ^b^	0c	0c
24 h of growth	28.85 ± 3.75 ^a^	35.28 ± 3.03 ^b^	1.71 ± 0.14 ^c^ (5.9 ± 0.48%of resistance)	0 ^d^
48 h of growth	27.96 ± 3.6 ^a^	21.37 ± 1.69 ^b^	13.39 ± 0.75 ^c^ (47.88 ± 2.68% of resistance)	3.98 ± 0.2 ^d^ (14.23 ± 0.64% of resistance)
96 h of growth	25.04 ± 3.07 ^a^	21.71 ± 1.14 ^a^	13.26 ± 0.97 ^b^ (52.95 ± 3.86% of resistance)	14.21 ± 0.72 ^b^ (65.45% ± 3.3 of resistance)
168 h of growth	7.17 ± 0.38 ^a^	4.38 ± 0.38 ^b,^*	2.29 ± 0.1 ^c^ (31.88 ± 1.43% of resistance)	1.68 ± 0.04 ^d^ (38.36 ± 0.92% of resistance)

^a^ Mean ± confidence level (*p* = 0.05). Values in the same rows followed by different letters are significantly different at *p* < 0.05 by t-test. a, b, c—0.05 > *p* > 0.005.; d—did not differ significantly. The incubation medium for the experiments contained 50 mM KP_i_, pH 5.5; and 1% glucose. Values are mean ± S.E.M from 5–6 independent experiments.

## Supplementary Materials

The following are available online at https://www.mdpi.com/2076-2607/8/1/91/s1.
