# Supplementary material for: Metabolic Remodeling during Long-Lasting Cultivation of the Endomyces magnusii Yeast on Oxidative and Fermentative Substrates
Source: Microorganisms. 2020 Jan 9;8(1):91. doi: 10.3390/microorganisms8010091 (PMC7022524; doi:10.3390/microorganisms8010091)
Supplement: Supplementary file 1 [file microorganisms-08-00091-s001.pdf]

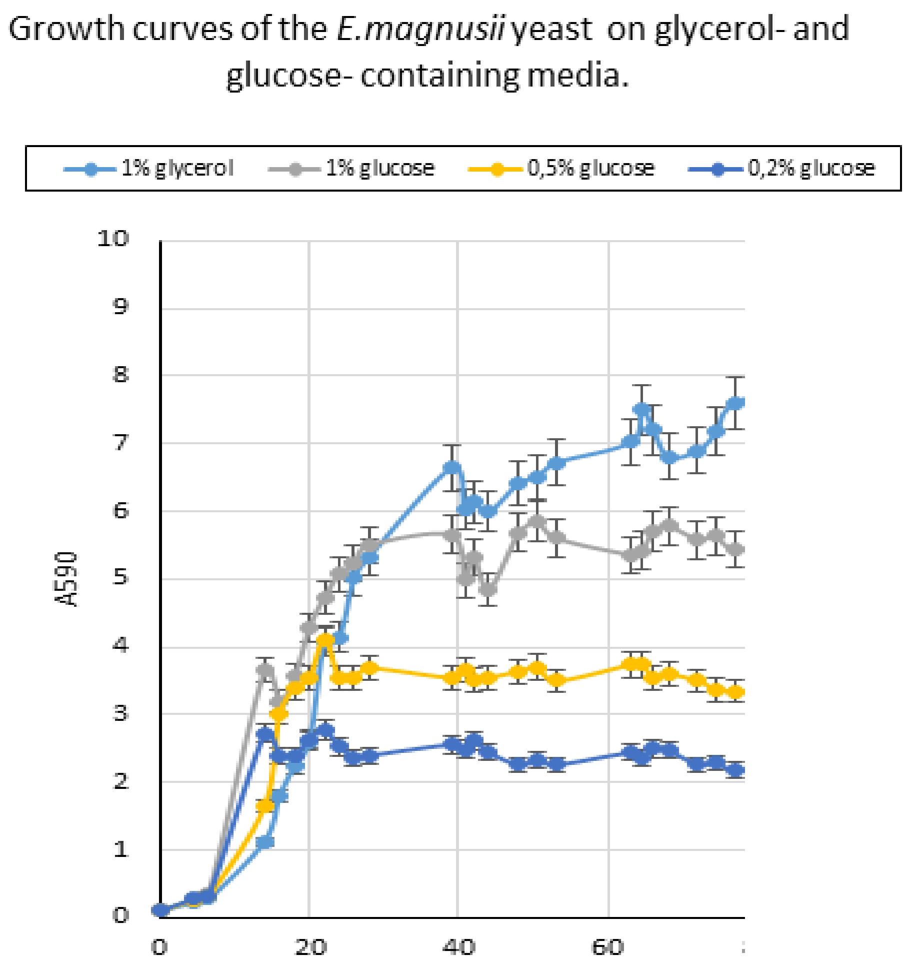

FIGURE S1 Growth curves of the *E.magnusii* yeast on glycerol- and glucose-containing media.

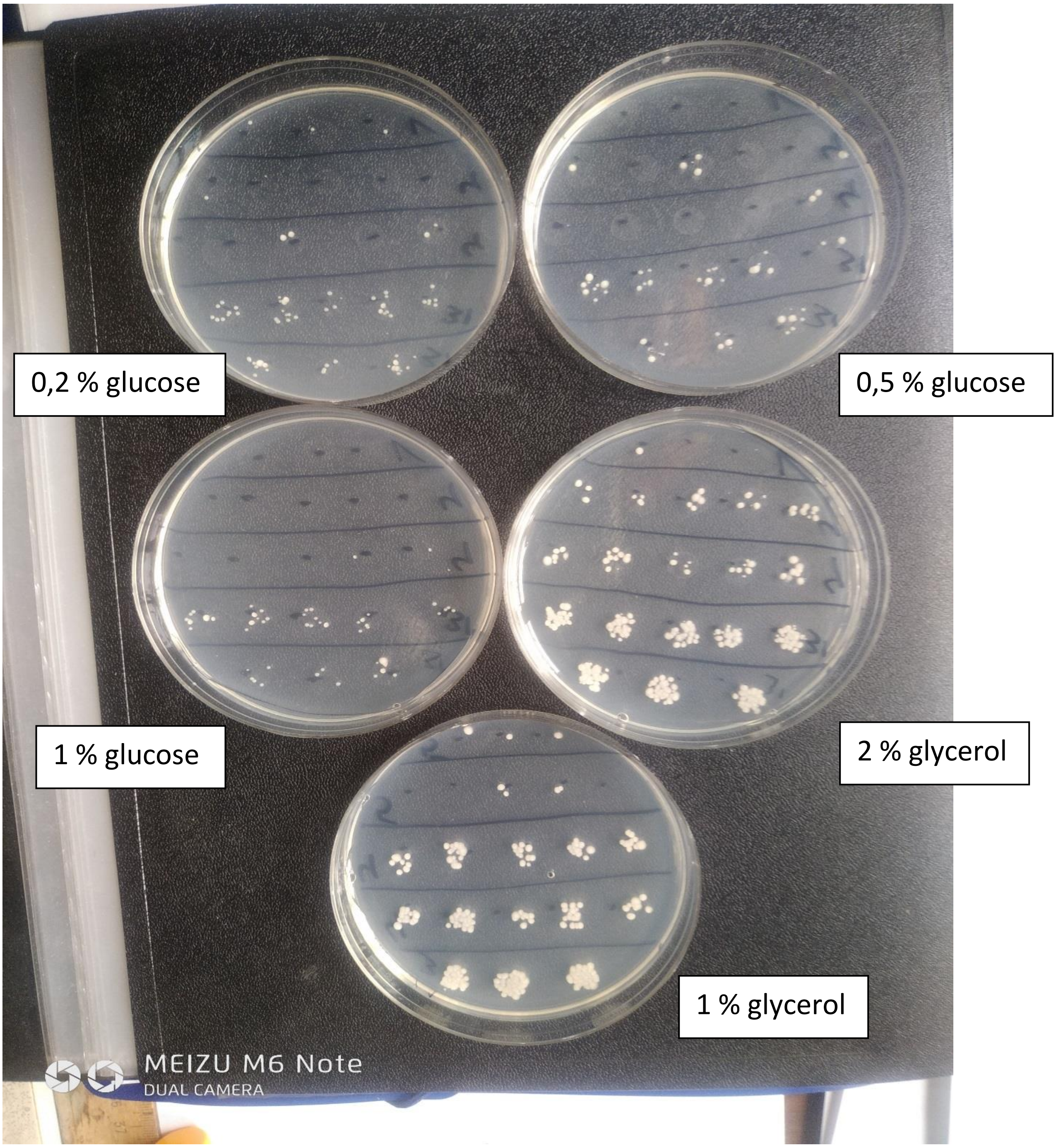

FIGURE S2. The survival of the yeast grown in glycerol- and glucose-containing media in 4 weeks.
